# Sub-threshold depression and antidepressants use in a community sample: searching anxiety and finding bipolar disorder

**DOI:** 10.1186/1471-244X-11-164

**Published:** 2011-10-10

**Authors:** Mauro G Carta, Leonardo Tondo, Matteo Balestrieri, Filippo Caraci, Liliana dell'Osso, Guido Di Sciascio, Carlo Faravelli, Maria Carolina Hardoy, Maria E Lecca, Maria Francesca Moro, Krishna M Bhat, Massimo Casacchia, Filippo Drago

**Affiliations:** 1 Department of Public Health, University of Cagliari, Cagliari, Italy; 2 Department of Psychiatry, Harvard Medical School, Boston, Massachusetts, USA; 3 Inter-University Center for Behavioural Neurosciences, DPMSC, University of Udine, Udine, Italy; 4 Department of Drug Sciences, University of Catania, Italy; 5 Department of Psychiatry, Neurobiology, Pharmacology and Biotechnology, University of Pisa, Pisa, Italy; 6 Department of Neurological and Psychiatric Sciences, University of Bari, Bari, Italy; 7 Department of Neurology and Psychiatry, Florence University, Firenze, Italy; 8 Department of Psychiatry, Reald University, Vlore, Albania; 9 Department of Neuroscience and Cell Biology, University of Texas Medical Branch, Galveston, Texas, USA; 10 Department of Science of Health, University of L'Aquila, L'Aquila, Italy

## Abstract

**Background:**

To determine the use of antidepressants (ADs) in people with sub-threshold depression (SD); the lifetime prevalence of mania and hypomania in SD and the link between ADs use, bipolarity and anxiety disorders in SD.

**Methods:**

Study design: community survey. Study population: samples randomly drawn, after stratification from the adult population of municipal records. Sample size: 4999 people from seven areas within six Italian regions. Tools: Questionnaire on psychotropic drug consumption, prescription; Structured Clinical Interview NP for DSM-IV modified (ANTAS); Hamilton Depression Rating Scale (HAM-D); Mood Disorder Questionnaire (MDQ); Short Form Health Survey (SF-12). SD definition: HAM-D > 10 without lifetime diagnosis of Depressive Episode (DE).

**Results:**

SD point prevalence is 5.0%. The lifetime prevalence of mania and hypomania episodes in SD is 7.3%. Benzodiazepines (BDZ) consumption in SD is 24.1%, followed by ADs (19.7%). In SD, positive for MDQ and comorbidity with Panic Disorder (PD) or Generalized Anxiety Disorders (GAD) are associated with ADs use, whereas the association between a positive MDQ and ADs use, without a diagnosis of PD or GAD, is not significant. Only in people with DE the well-being (SF-12) is higher among those using first-line antidepressants compared to those not using any medication. In people with SD no significant differences were found in terms of SF-12 score according to drug use.

**Conclusions:**

This study suggests caution in prescribing ADs to people with SD. In people with concomitant anxiety disorders and SD, it should be mandatory to perform a well-designed assessment and evaluate the presence of previous manic or hypomanic symptoms prior to prescribing ADs.

## Background

Patients with mild-to-moderate, chronic or episodic dysthymia and anxiety may not benefit greatly from antidepressant treatment [1]. According to this study, antidepressants have limited short-term efficacy in unipolar depressive disorders and even less in acute bipolar depression. Moreover their long-term prophylactic effectiveness in recurrent unipolar major depression remains uncertain and is doubtful in bipolar depression [1]. These limitations may, in part, reflect the excessively broad concept of unipolar depression. The current subtyping of depression is based on the DSM-IV-TR categorical division of bipolar and depressive disorders. Current evidence, however, supports a dimensional approach to depression, as a continuum/spectrum of overlapping disorders, ranging from bipolar I depression to major depressive disorder [2].

Treatment-refractory depression may reflect failure to distinguish depressive conditions, particularly in bipolar disorder, that are inherently less responsive to antidepressants. Antidepressants probably should be avoided in bipolar depression, mixed manic-depressive states, and in neurotic depression. Expectations of beneficial effects of antidepressant treatments for specific types of patients with symptoms of depression or anxiety require critical re-evaluation.

Taking into account the hypothesis that in sub-threshold depressions (SDs) the use of antidepressants may be ineffective, this study aims to examine this hypothesis in a large community sample:

1) The amount of use of antidepressants in people with depressive symptoms (HAM-D > 10) without lifetime diagnosis of Depressive Episode (DE).

2) The lifetime prevalence of mania and hypomania in such subjects.

3) The role of anxiety disorders in the use of antidepressants in people with SD.

4) The link between antidepressants use, bipolarity and anxiety disorders in SD.

5) Whether or not subjects with SD treated with antidepressants actually need to be treated.

## Methods

### Design

The study is a community survey based on the database of the National Italian Survey "The use of antidepressants in Italy" [3]. Face-to-face interviews were carried out at candidates' homes.

### Recruitment methods and study sample

The study sample was randomly drawn from the adult population of municipal records in seven different areas from different Italian locations with wide variations in socio-economic conditions: [a] Sicily (Catania), [b] Sardinia (Iglesias), and [c] Puglia (Bari) in southern Italy; [d] Abruzzo (L'Aquila) and [f] Tuscany (Florence and Pisa) in central Italy; [g] Friuli-Venezia Giulia (Udine) in northern Italy. In each area, both urban and rural sub-areas were selected. The sample of Florence (Sesto-Fiorentino) was only urban and Pisa was only rural. A third of the sample in each centre was drawn from three variously populated municipalities; less than 2,000, from 2,001 to 10,000 inhabitants, and from 10,001 to 20,000 inhabitants.

Randomization was performed after stratification by sex and four different age groups (18-24; 25-44; 45-64; > 64).

Using the above mentioned methodology, a sample of 4999 people was drawn from the seven areas. The size of the subsamples were: 704 in L'Aquila; 971 in Bari; 666 in Catania; 846 in Florence; 465 in Iglesias; 464 in Pisa, and 882 in Udine.

Subjects were contacted at home by phone or by mail by the local coordinator of the study.

### Interview, tools and study assessment

Interviews were carried out using the following tools:

1. Ad-hoc form to assess basic demographic data and psychotropic drug consumption, prescription circumstances and health-services utilization previously used and validated in a regional survey [4]. For all the subjects, consumption of antidepressant drugs was ascertained. Positive use was identified as subjects consuming antidepressant drugs at therapeutic dosages for every day for at least 15 days prior to the interview.

2. The "Advanced Neuropsychiatric Tools and Assessment Schedule" (ANTAS), a computerized semi-Structured Clinical Interview derived from the non patient version (SCID-I/NP) for DSM-IV [5] to assess the presence of psychiatric disorders (this, as stated in the study protocol, requires a clinical competence to administer). A reliability study of the diagnosis derived from the ANTAS compared to SCID has been carried out and the results have been previously published [6]. The results for mood and anxiety diagnosis with SCID showed a mean *k *of 0.85 [3,6].

3. The Mood Disorder Questionnaire (MDQ) ([7], Italian version [8]) for the assessment of bipolar spectrum disorders.

4. The depressive symptoms were measured by means of Hamilton Depression Rating Scale (HAM-D), [9].

5. Quality of life was evaluated with the Short Form Health Survey (SF-12) [10]. The SF-12 includes the following dimensions: physical activity, physical health limitations on role or activities, emotional state, physical pain, self-evaluation of general state of health, vitality, social activity and mental health. The period of measurement is the previous month. Highest scores correspond to better conditions and quality of life.

For the purpose of the present study we used these definitions:

1. SD = people having, at the time of the interview, depressive symptoms (HAM-D score > 10) without an ANTAS-SCID-DSM-IV lifetime diagnosis of Depressive Episode.

2. Depressive Episode (DE) point prevalence = people with an ANTAS-SCID-DSM-IV Depressive Episode diagnosis at the time of the interview; they may have a lifetime ANTAS diagnosis of Major Depressive Disorder (MDD) or Bipolar Disorder (BD).

3. Not Depressed (ND) people = people not showing any relevant depressive symptoms at the time of the interview (HAM-D score < 10).

4. Lifetime diagnosis of manic-hypomanic episode in SD = people having MDQ lifetime positivity (score > 7).

All the people interviewed in the community were included in the analysis.

### Interviewers and training

Interviewers were selected from psychologists and physicians with at least two years of experience in clinical psychiatric work following graduation.

They received a common and intensive training in the use of the research instrument and administration of home interviews by the Coordinating Unit.

Interviewers were provided with a laptop computer and software to record data during the interview.

Two assistant researchers from the Coordinating Unit traveled to each field unit and interviewed at least seven patients and three control subjects who had been interviewed by the local interviewers. Differences in results were discussed and resolved. The diagnosis reliability between coordinator researchers and each unit had a *k *mean higher than 80.

### Monitoring and Quality Control

Interview quality was monitored by cross-examining the interviewers every three months and having at least 120 interviews repeated by different interviewers.

### Sample Size

It was envisaged that from 60% to 65% of the original sample (a planned sample of at least 4,800 interviews) would take part in the survey (5% of members were expected to be deceased or moved, 10% were expected to be non retrievable and 20% were considered the refusal rate) for an expected total of about 3,000 interviewed subjects. This sample size was expected to provide a 95% confidence interval of ± 0.036% of the expected prevalence summary estimate of 4% of both antidepressant consumption and bipolar disorders as MDQ positives (relative standard error being around 7%). This sample was also planned for recording SD (main objective of the present study), considering that the expected point prevalence of SD was 6-9%. This is because, the ECA study of Judd and Coll [11] found a prevalence of 11.8% using a less inclusive diagnosis. A similar frequency was also found in the Zurich study by Angst and Coll [12].

### Ethical Aspects

An informed consent for the use of anonymous data suitable for an aggregate study was signed by each candidate. The study was approved by the ethical committee of the Italian National Health Institute (Rome). Data were not nominal at source and each subject was identifiable with a code number.

## Results

The characteristics of the enrolled sample by age, sex and the rate of non-interviewed have been already published [3]. No significant statistical differences were found between the interviewed sub-samples and the randomized sub-samples according to age and sex [3].

A total sample of 3389 subjects was enrolled (57.7% female).

Table 1 shows the prevalence of SD by sex in the total sample. At the time of interview, 5.0% of the sample had SD. SD is more frequent in females than in men (5.5% vs 2.0%, OR = 2.86 CL95% 1.4-4.3). The prevalence of lifetime mania and hypomania episodes in people with SD is 7.3% (6.5 in females, 10.5 in males). The table shows that the prevalence of mania and hypomania in SD is lower than in people with DE but higher than in the overall sample without depressive symptomatology (SD or DE) at the time of the interview.

**Table 1:** Prevalence of Sub-threshold Depression (SD) (HRDS > 10 without criteria for lifetime Depressive Episode) by sex and frequency of Mania and Hypomania detected by MDQ in SD, Depressive Episode point prevalence = DE and in the overall sample without depressive depression (SD or DE) = ND, by sex

	Male	%	Female	%	Total	%	OR (F)	CL 95%	Χ2 1df	P
**SD**	29	2.0	108	5.5	137	5.0	2.86	1.4-4.3	25.5	< 0.0005

**1. MDQ in SD**	3	10.3	7	6.5	10	7.3	0.6	0.24-1.39	0.10	0.76

**2. MDQ in DE**	4	23.5	11	28.9	15	27.3	1.3	0.41-2.10	0.01	0.92

**3. MDQ in ND**	42	3.0	36	2.0	78	2.4	0.6	0.34-1.05	3.14	0.08

**MDQ in the overall sample**	49	3.4	54	2.7	103	3.0	0.8	0.5-1.2	1.00	0.312

**Χ2 by columns** **3df**	25.7		107.0		100.4					

**P**	0.0001		0.0001		0.0001					

**Homogeneity by cells in columns (**1DF)**:**

**Column Male **1vs2, χ2 = 0.6, P = 0.43; 1vs3, χ2 = 2.86, P = 0.09; 2vs3 χ2 16.3, P < 0.001; **Column Female**1vs2, χ2 = 11.1, P < 0.001; 1vs3, χ2 = 7.05, P = 0.006; 2vs3 χ2 = 98.8, P < 0.0001; **Column Total **1vs2, χ2 = 12.5, P < 0.001; 1vs3, χ2 = 6.4, P < 0.01; 2vs3 χ2 = 91.2, P < 0.0001.

Table 2 shows the psychoactive drugs and other treatments consumed by people with SD in the overall sample and by sex. Benzodiazepines (BDZ) are the drugs with highest consumption (24.1%) followed by antidepressants (19.7%). First line antidepressants (including SSRI, TCA, IMAO, SNRI, NaSSA and Bupropion) are used by 14.6% of the sample and second line antidepressants (low-dosage Benzamides, low-dosage Quetiapine, Trazodone, Nefazodone, Adenosin-methionine and Hypericum Perforatum) by the remaining 5.1%. We note that low-dosage Benzamides are available for minor depression only in Italy. Psychotherapies are well represented (9.5%) and homeopathic treatments are limited (2.9%). The use of antidepressants and benzodiapines is more frequent among females with SD than among males with SD, but the difference is not statistically significant.

**Table 2:** Psychoactive drugs and other treatments in Sub-threshold Depression (HRDS > 10 without lifetime criteria for Depressive Episode) by sex

Treatment	Males	%	Females D	%	Total (males and females)	%	OR (F)	CL 95%	Χ2 1df	P
**First-line Antidepressants°**	2	6.8	18	16.6	20	14.6	2.7	0.1-96.4	1.05	0.30

**Second-line Antidepressants§**	1	3.4	6	5.6	7	5.1	1.6	0.1-31.2	0.1	0.98

Benzodiazepines*	7	24.1	33	30.6	40	24.1	1.4	0.2-6.3	0.2	0.66

**Homeopathics**	1	3.4	3	2.7	4	2.9	0.8	0.3-2.1	0.2	0.68

**Psychotherapy**	3	10.3	10	9.2	13	9.5	0.9	0.4-2.0	0.1	0.86

°SSRI, TCA, IMAO, SNRI, NaSSA, bupriopion; § low-dosage benzamides, low-dosage quetiapine, trazodone, nefazodone, Adenosin-methionine, Hypericum Perforatum

When we compare the use of psychoactive drugs and other treatments in the overall sample, in ND subjects, subjects with DE point prevalence and in subjects with SD (Table 3), the frequencies of use of first-line and second-line antidepressants rank as follows: subjects with DE > STD > ND. Treatments with BDZ and psychotherapy do not show any difference between DE and STD, but these two groups show higher use than ND people. Homeopathic treatments seem to be typically used in SD with a statistically significant difference only when compared to ND people.

**Table 3:** Comparison of psychoactive drugs use and other treatments in Sub-threshold Depression (HRDS > 10 without criteria for lifetime Depressive Episode) = SD; subjects with Depressive Episode point prevalence = DE and in the overall sample without Depression (SD or DE) = ND

	2. SD	3. DE	1. ND	Overall Sample	Χ2 2DF	P
Total	137 (4.0)	55 (1.6)	3206 (94.3)	3398		

°First line Antidepressants	20 (14.6)	17 (30.9)	69 (2.1)	106 (3.1)	210.2	0.0001

§Second line Antidepressants	7 (5.1)	14 (25.4)	32 (1.0)	53 (1.6)	14.7	0.0001

*Benzodiazepines	40 (29.1)	14 (25.4)	188 (5.8)	242 (7.1)	147.9	0.0001

°°Homeopathics	4 (2.9)	0	21 (0.6)	25 (0.7)	8.1	0.018

**Psychotherapies	13 (9.5)	8 (17.8)	50 (1.5)	71 (2.1)	95.3	0.0001

**°**Homogeneity by cells: 1vs2, χ2 = 74.1, 1DF P < 0.0001; 1vs3, χ2 = 205.7, 1DF P < 0.0001; 2vs3 χ2 = 9.8, 1DF 0.002; **§**Homogeneity by cells: 1vs2, χ2 = 12.5, 1DF P < 0.0001; 1vs3, χ2 = 76.3, 1DF P < 0.0001; 2vs3, χ2 = 14.6, P < 0.0001; 2vs3 χ2 = 5.6, 1DF P = 0.018; * Homogeneity by cells: 1vs2, χ2 = 109.4, 1DF P < 0.0001; 1vs3, χ2 = 44.5, 1DF P < 0.0001; 2vs3 χ2 = 0.1, 1DF NS; °° Homogeneity by cells: 1vs2, χ2 = 4.6, 1DF P < 0.031; 1vs3, χ2 = 0.1, 1DF NS; 2vs3 χ2 = 0.3, 1DF NS; ** Homogeneity by cells: 1vs2, χ2 = 40.4, 1DF P < 0.0001; 1vs3, χ2 = 45.0, 1DF P < 0.0001; 2vs3 χ2 = 0.5, 1DF NS

No demographic factors (sex, age, geographic distribution) appear to be of relevance in the frequency of prescription of antidepressants in the SD sample (Table 4). Of relevance, in people with SD, a positive MDQ diagnosis is associated with antidepressant use (the calculation was carried out after standardizing for sex and age).

**Table 4:** Determinants of all ADs (first and second line) prescriptions in Sub-threshold Depression (HRDS > 10 without criteria for lifetime Depressive Episode)

	N(%)	OR	95% CI	χ2	P
Females	24 (22.2)	2.4	0.5-10.6	1.3	0.244

North-Center	20 (23.8)	2.0	0.7-6.0	1.7	0.194

Age 18-24	2 (10.0)	1.01	0.95-1.1	0.1	0.6

Age 25-64	19 (17.4)	0.5	0.9-1.3	1.1	0.291

Age > 64	6 (33.3)	2.3	0.6-8.8	1.5	0.214

*MDQ > 7	**7.5 (41.7)**	**3.6**	**1.1-40.5**	**4.8**	**0.028**

*Comorbidity PD	**9.6 (53.3)**	**17.2**	**6-49.2**	**27.2**	**0.0001**

*Comorbidity DOC	2.8 (40.0)	2.9	0.5-1.3	0.8	0.369

*Comorbidity GAD	**6.9 (43.1)**	**4.2**	**1.2-13.8**	**5.6**	**0.018**

*Standardized by age, sex and residence (N/S); °Vs overall sample receiving ADs (with exclusion of DE point prevalence)°°The Odds Ratio is calculated vs all other age frequencies

Comorbidity with PD or GAD is also associated with the use of antidepressant drugs. Re-calculating the weight of the association between MDQ positivity and antidepressants use, standardizing by having a diagnosis of PD or GAD, the significance of association disappears (χ^2 ^= 1.6 (1df), *p *= 0.2, OR = 2.6). Thus, the basis for taking antidepressants is the anxiety disorder and MDQ positivity is probably occurring as a confounding factor.

Table 5 analyzes the well being of the subjects in the sample, measured with SF-12, according to the diagnosis and the treatment of BDZ and antidepressants. In the community, only in people with DE the well-being is higher in those using first-line antidepressants compared to those not using any medication. Moreover, no differences are observed for the second-line antidepressants or BDZ. In people with SD no differences are found even for first-line antidepressant.

**Table 5:** Well being (SF-12) by treatment: SD, Sub-threshold Depression (HRDS > 10 without criteria for lifetime Depressive Episode); DE, Depressive Episode point prevalence; Total Depressive Symptoms = SD plus DE

	First line AD	Second line AD	Only BDZ	No AD or BDZ	F	P
**°SD**	30.8+/-4.5 (n = 20)	30.4+/-4.3 (n = 7)	28.3+/-4.3 (n = 35)	29.1+/-5.1 (n = 75)	(DF 3,133, 136) 1.3	0.288

*DE	31.6+/-5.6* (n = 17)	30.3+/-4.9 (n = 14)	28.6+/-5.1 (n = 9)	25.8+/-5.1* (n = 15)	(DF 3,51,54) 1.8	0.002

§Total depressive symptoms	31.2+/-5.0 (n = 37)	30.3+/-4.7 (n = 21)	28.4+/-4.5 (n = 44)	28.5+/-5.1 (n = 90)	(DF 3, 188,191) 3.0	0.0019

T test Bonferroni 1vs4, 51 DF zP < 0.05, Not Significant differences in the other comparisons; °t test Bonferroni 133 DF, No significant differences; § t test Bonferroni 188 DF, No significant differences

## Discussion

The study indicates that around 5% of people in the community have SD and that SD is higher in women than in men (5.5 vs 2%). People with SD have a lifetime prevalence of manic and hypomanic episodes detected by MDQ (7.3%). This frequency falls in the middle, between people without depression (DE or SD) at the time of interview (2.4%) and people with DE (27.3%). If we consider that SD is more prevalent than DE in the community (5% vs 2.4%), the relevance of MDQ positivity at the community level is quite similar in DE (MDQ positivity 0.4% of the whole community sample) and in SD (MDQ positivity 0.3% of the sample). This is an interesting point because it is intuitive that the identification of a previous manic or hypomanic episode should be easiest in people with a clear diagnosis of DE.

For the purpose of this study we decided to compare the lifetime prevalence of MDQ Positivity in people showing symptoms of DE without a lifetime diagnosis of DE and in people with DE at the time of interview. Our purpose is to define how much is of clinical and public health relevance if a person with depressive symptoms at the time of a hypothetical clinical contact but without prior diagnosis of depressive episode has manic or hypomanic lifetime episode and how much this phenomenon may be related with the antidepressants use. In this sense the definition of SD as people having a HAM-D score of > 10 is an operational choice for detecting people without diagnosis of DE but with a relevant depressive symptomatology that may result in antidepressant prescription.

We take into account that the diagnosis of Mania and Hypomania by SCID may be too restrictive [13] and we also use the MDQ positivity to measure the "bipolarity spectrum". The under recognition of BD in people with DE is not the specific objective of this study; this topic has been addressed in another study using the same database [14].

The results of our community study show that people with SD use largely the first- (14.6%) and the second-line antidepressants (5.1%), as well as BDZ (24.1%). The consumption of antidepressants is similar in people with SD and DE, only smaller for the first-line antidepressants but higher for all other medications than in people without depressive symptoms. The use of antidepressants in SD is more significant with comorbidity factors PD or GAD as per treatment guidelines, indicating antidepressants as the first-line of treatment for PD [15,16] and GAD [17].

Nevertheless in our community sample, the diagnoses of PD or GAD are strictly associated with MDQ positivity. This is expected given the recent findings in a recent study [18] determining any confounding relationship between MDQ positivity and antidepressant use in people with SD. In fact, antidepressants are frequently used in the sub-sample with a previous manic or hypomanic episode, detected by MDQ, with a high risk for developing BD (even without a DE).

Depressive episodes are the most prevalent component in bipolar disorders, even when, as in a community, they are showing a clinical picture not meeting criteria for DE. There is a growing awareness that treatment of bipolar depression is one of the greatest challenges in modern psychiatry [19,20] since response to antidepressants is often unsatisfactory despite the overuse of these agents [19]. Other studies also suggest that treating bipolar depression with antidepressants is likely a bad practice [20,21]. There is indeed a strong rationale for a cautious approach to antidepressant use in bipolar disorder [18,22], which is based on the following findings:

(i) The risk of antidepressant-induced mood-cycling is high [23].

(ii) Antidepressants have not been shown to definitively prevent suicides and reduce

mortality, unlike long-term lithium treatment [24].

(i) Antidepressants have been shown less effective than mood stabilizers or atypical antipsychotics in acute bipolar depression and less effective than mood stabilizers in preventing depressive relapse in BD. A recent systematic review and meta-analysis, reexamining the efficacy and safety of antidepressants use for acute treatment of bipolar depression, found antidepressants not statistically superior to placebo or other current standard treatment for bipolar depression [25].

European guidelines exert a more flexible attitude towards the use of antidepressants, whereas currently published American guidelines explicitly do not recommend antidepressants in the treatment of bipolar depression, unless the depressive episode is severe [26]. Our study indicates that antidepressants are broadly used (20% of people) in a large community subsample with SD and is strictly associated with bipolar disorders with the possible induction of mood-cycling in the sub-sample.

Question arises as to whether or not subjects with SD treated with antidepressants need to be further treated. This issue requires some examination of our results in light of results reported in recent literature. First, according to the hypothesis proposed by Ghaemi [1], our study indicate that the antidepressant use does not appear to be associated with improvement in the quality of life of people with SD; this is in contrast to people with DE in whom the use of first-line of antidepressants appears to be associated with a better quality of life. This finding needs to be confirmed by further studies, nonetheless, the results are in agreement with the findings of a recent review of clinical randomized trials, which suggest that there is no clinically significant difference between antidepressants and placebo in patients with minor depression [27].

Second, subject with SD and positivity for MDQ include: a) people with manic episode, fulfilling the criteria for Bipolar Disorders; people with hypomanic episode (not meeting the criteria for manic episode) including, b) people with cyclothymic disorder (the essential features of cyclothymic disorder is a chronic, fluctuating mood disturbance involving numerous periods of hypomanic and depressive symptoms), and/or c) people with hypomanic episode who do not meet the criteria for cyclothymic disorder (they should be classified as Bipolar Disorder Not Otherwise Specified).

As with Bipolar Disorder, use of antidepressants in cyclothymic disorder is typically not recommended, unless they're combined with a mood stabilizer. As with bipolar disorder, taking antidepressants alone can trigger potentially dangerous manic episodes. Patients with cyclothymia may switch to type II illness when treated with antidepressants [28]. Some evidence indicates that cyclothymia may be also associated with high risk for switch to rapid cyclicity [29]. Concerning the risk about switch to mania in people with SD and singular/isolated episode of hypomania using antidepressants, some studies suggest a similar risk in Bipolar Disorder Not Otherwise Specified than in other Bipolar Disordes [30]. Thus, people with SD and mania/hypomania detected by MDQ positivity are in a group where the use of antidepressants alone is counterindicated. In our study, this sub-sample is represented by 7.3% of the total SD in the community.

Third, a strong association between MDQ positivity, SD and anxiety disorders as Panic Disorder and Generalized Anxiety Disorders was found in our study. In these Anxiety disorders, antidepressants are an efficacious treatment and SSRI, as previous cited, are indicated as the first choice for treatment as per most treatment guidelines for anxiety disorders [15-17,31]. Nevertheless it was be found that co-morbidity with anxiety spectrum disorders and anxiety disorders is associated with rapid switching [32], thus the use of AD alone (without mood stabilizers) in people with SD, MDQ positivity and anxiety disorders may be an option to be considered with extreme caution. Therefore, the use of antidepressants in SD and Anxiety disorders must be preceded by an efficacious screening for mania/hypomania.

Fourth, a large number of studies have found that counseling and psychosocial therapies in sub threshold depression is highly effective, at least in the short-term [33]. Similarly a brief psychological therapies were effective for anxiety [33]. Thus, antidepressants do not appear to have significant advantages for treating SD, and, it may even be dangerous if used in people with SD and MDQ positivity. Our results, together with the results of the recent above cited systematic review [27], suggest that antidepressants should not be considered for treatment of individuals with SD.

### Limitations of the study

Our study has some significant limitations: first, some of the phenomena observed have a very low prevalence despite the use of a large sample of around 4,000, thus the results need to be considered with caution and confirmed by additional surveys. Second, the observational design is ineffective to account for people's well being related to a treatment; in this perspective a community survey have to be considered only as a source of hypothesis and these specific results must be considered only as an heuristic contribute. Third, the sample is representative of certain areas in Italy and is not representative of the nation as a whole.

Finally, serious doubts have been raised on the ability of the MDQ to detect milder bipolar disorders [34]. As a matter of fact, according to Zimmerman et al [35] MDQ shows low sensitivity in clinical settings among cases affected by bipolar disorder. The cross-national validation studies of MDQ reported good accuracy in the UK [36], Turkey [37] and Spain [38] and less encouraging results in France [39]. The validation of the Italian version of MDQ in the general population [3] revealed fairly good accuracy, with sensitivity of 0.70, specificity of 0.87, PPV of 0.47 and NPV of 0.95 (using a cut-off of seven, as in the present study). Moreover, the comparison of the MDQ with another screening instrument for Bipolar disorder (Hypomania Checklist (HCL-32)] showed high consistency [40]

## Conclusions

Our study appears to suggest a greater caution in prescribing antidepressants to people with SD, especially antidepressants with higher risk to induce mania, particularly to people with concomitant anxiety disorders. In SD, a well-designed assessment to evaluate the presence of previous manic or hypomanic symptoms should be mandatory before prescribing antidepressants.

## Competing interests

The authors declare that they have no competing interests.

## Authors' contributions

MGC participated in the design and coordination of the study, in the acquisition and analysis of the data and drafted the manuscript. LT participated in the analysis of the data and drafted the manuscript. MB, FC, LdO, GdS, CF participated in the design of the study, in the acquisition and analysis of the data and drafted the manuscript. MCH participated in the design and coordination of the study. MFM and MEL participated in acquisition of data and critical revision of the manuscript. KMB participated in the analysis of the data and drafted the manuscript. MC and FD participated in the design of the study, in the acquisition and analysis of the data and drafted the manuscript. All authors read and approved the final manuscript.

## Pre-publication history

The pre-publication history for this paper can be accessed here:

http://www.biomedcentral.com/1471-244X/11/164/prepub
